# Phonon Raman spectra of colloidal CdTe nanocrystals: effect of size, non-stoichiometry and ligand exchange

**DOI:** 10.1186/1556-276X-6-79

**Published:** 2011-01-12

**Authors:** Volodymyr Dzhagan, Irina Lokteva, Cameliu Himcinschi, Xiaoping Jin, Joanna Kolny-Olesiak, Dietrich RT Zahn

**Affiliations:** 1 V. E. Lashkaryov Institute of Semiconductors Physics, National Academy of Sciences of Ukraine, prospekt Nauky 45, Kyiv, 03028, Ukraine; 2 Energy and Semiconductor Research Laboratory, Department of Physics, Carl von Ossietzky University of Oldenburg, Carl-von-Ossietzky Str. 9-11, Oldenburg, 26111, Germany; 3 Institute of Theoretical Physics, TU Bergakademie Freiberg, Leipziger Str. 23, Freiberg, 09596, Germany; 4 Semiconductor Physics, Chemnitz University of Technology, Reichenhainer Str. 70, Chemnitz, 09107, Germany

## Abstract

Resonant Raman study reveals the noticeable effect of the ligand exchange on the nanocrystal (NC) surface onto the phonon spectra of colloidal CdTe NC of different size and composition. The oleic acid ligand exchange for pyridine ones was found to change noticeably the position and width of the longitudinal optical (LO) phonon mode, as well as its intensity ratio to overtones. The broad shoulder above the LO peak frequency was enhanced and sharpened after pyridine treatment, as well as with decreasing NC size. The low-frequency mode around 100 cm^-1 ^which is commonly related with the disorder-activated acoustical phonons appears in smaller NCs but is not enhanced after pyridine treatment. Surprisingly, the feature at low-frequency shoulder of the LO peak, commonly assigned to the surface optical phonon mode, was not sensitive to ligand exchange and concomitant close packing of the NCs. An increased structural disorder on the NC surface, strain and modified electron-phonon coupling is discussed as the possible reason of the observed changes in the phonon spectrum of ligand-exchanged CdTe NCs.

PACS: 63.20.-e, 78.30.-j, 78.67.-n, 78.67.Bf

## Introduction

Among semiconductor nanocrystals (NCs), colloidal CdTe NCs attract increasing interest due to their large exciton Bohr radius (7.3 nm), pronounced quantum size effect, optical activity both in the visible and near-infrared spectral ranges (with a bulk band gap of 1.475 eV) and chemical flexibility at the NC surface. These properties make the CdTe NСs promising for various optoelectronic and biological applications: light-emitting diodes, living cells' fluorescent labels, photovoltaic devices etc. [1-5]. Since as-synthesized colloidal NCs are usually stabilized by an organic ligand shell, inhibiting an efficient charge transfer out of the NC, a crucial step towards realizing some applications is the removal of these ligands from the NC surface and/or its exchange for other functional molecules [1,2,6,7]. Previously, the removal of the surface ligands (oleic acid (OA) and tri-*n*-octylphosphine (TOP)) from solution processed CdTe NCs by a straightforward pyridine treatment was found to drastically reduce the photoluminescence (PL) lifetime and quantum yield [8]. No significant broadening of the absorption or PL features and Stokes shift value being observed upon pyridine treatment in [8] indicated that the quality of the NC surface is not much deteriorated.

Though phonons play a crucial role in the dynamics of charge carriers and significantly determine the stationary optical properties of NCs [1], detailed studies of the vibrational properties of CdTe NCs are rather rare [9-12]. The phonon spectra, electron-phonon coupling and some properties of the electronic states can be studied by resonant Raman scattering (RRS) spectroscopy [13]. In addition, the parameters of the phonon peaks in the RRS spectra provide information about the structure of NCs [9-31]. Nevertheless, the nature of phonon features of nanocrystalline semiconductors is not well understood at present. In addition to most frequently discussed effect of phonon confinement (PC) onto the parameters of the phonon peaks, there obviously exist other important factors which lead to discrepancies in the reports of different authors concerning the effect of NC size onto the phonon spectrum. In particular, matrix-induced stress and zinc in-diffusion were proved to have noticeable effect on interpretation of the phonon spectra and their size dependence for glass-embedded NCs [23,30]. For colloidal NCs, the possible factors, competing with PC, which can have influence on the phonon spectrum, are yet to be studied. In several studies, a noticeable strain was revealed, which can originate from the surface tension of the NCs [32-34], while the effect of ligand is usually ignored. The only study of the ligand effect onto the longitudinal optical (LO) phonon in CdSe NCs [24] revealed noticeable tensile strain due to TOP(O) and hexadecylamine (HDA) ligands, while no ligand effect was found in [28] for a wide range of ligands/matrices. However, the result obtained in [24] may be in part due to using of *two different preparation routs *for TOPO- and HDA-passivated NC samples, as well as employment of treatment with pyridine of some (smallest) NCs in order to reduce the PL background in Raman spectra. In this work, the effect of oleic acid ligand exchange for pyridine was studied on the samples from *the same batch*, to elucidate the effect of the ligand onto vibrational properties of CdTe NCs. The coupling of different kinds of molecules to the NC surface atoms may cause noticeable strain in NCs due to re-arrangement (reconstruction) of the surface atoms [32-35], or by charge transfer between NC and molecule [31]. Raman spectroscopy on phonons has already proved to be an efficient tool of separating the factors of size, strain and chemical composition in different kinds of semiconductor nanostructures [9-31].

## Sample preparation and experimental setups

### Preparation of the CdTe NCs

All chemicals were used as received without further purification: cadmium oxide (CdO; 99%, Fluka), TOP (90%, Aldrich), OA (90%, Aldrich), 1-octadecene (ODE; Merck), tellurium powder (Te; purum p., Fluka), anhydrous pyridine (Normapur), dried *n*-hexane (AppliChem) and D-chloroform (99.8 at.% D, Aldrich).

Te precursor (TOPTe/ODE): A Te/TOP (10 wt.%) solution was prepared as follows: 4.5 g of TOP was added to 0.5 g (3.92 mmol) of Te in the glove box. This mixture was further heated to 200°C to 250°C under nitrogen flow till the dissolution of Te. Then, Te injection solution (TeTOP/ODE) was prepared by mixing 0.1460 g (0.114 mmol Te), 0.2910 g (0.229 mmol Te), 0.5104 g (0.4 mmol Te) or 1.021 g (0.8 mmol Te) of a Te/TOP solution with 2 ml of ODE to obtain the precursor ratio of Cd/Te = 7:1, 3.5:1, 2:1 and 1:1, respectively (Table 1).

**Table 1:** Summary of the main synthesis conditions, mean diameter, as well as parameters of the lowest excitonic of the NCs studied in this work

Sample	Cd/Te	Injection time^a^, s	Time of NCs growth, s	1st abs max (*E*_1_) in ODE/hex, nm/eV	NC *d*, nm (based on *E*_1_)
#621	7:1	5	10	621/2.00	3.9
#641	2:1	5	10	641/1.93	4.2
#642	1:1	20	10	642/1.93	4.2
#582	3.5:1	20	10	582/2.13	3.5

^a^Time period between the occurrence of Cd^0 ^precipitate and the injection of TeTOP.

Synthesis of high-quality CdTe NCs: A Cd precursor solution was prepared by mixing 0.1024 g of CdO (0.8 mmol) with 800 μl of OA (2.4 mmol) in 20 ml of technological-grade ODE in a single, three-neck, round-bottom flask. This mixture was heated to 100°C under vacuum for 30 min with permanent stirring. Later on, the system was turned to nitrogen flow and heated to 300°C, resulting in the formation of a homogeneous transparent solution of cadmium oleate Cd(OA)_2_. This solution of Cd-oleate complex in ODE was allowed to be boiled at 300°C without restriction, which led to the formation of a greyish precipitate of crystalline Cd^0 ^NCs. The TeTOP/ODE solution was injected at the time of 5 to 20 s after the Cd^0 ^appearance. The nanoparticle colloidal solution was cooled down to room temperature after the certain time of growth (Table 1).

Purification procedure: The solution of synthesized CdTe NCs was centrifuged to separate the Cd^0 ^particles. The extraction and precipitation procedure was adopted from [36]. Briefly, 20 ml of the original OA-coated CdTe nanocrystals (OD = 65) in ODE was diluted with 6 ml of hexane and extracted with 14 ml of methanol to remove the reaction by-products. The nanocrystals dispersed in the hexane-ODE layer were precipitated with excess acetone and methanol (5:1 ratio to the extracted hexane-ODE solution containing the nanocrystals). The nanocrystals were isolated through centrifugation and decantation. The precipitation step was repeated a couple of more times, and the final nanocrystal pellet on the bottom of the vial was redissolved in a desired solvent (hexane or pyridine).

Pyridine treatment: For ligand exchange, purified (as written above) CdTe NCs were redissolved in 7 ml of pyridine. Then the solution was heated to 65°C for 3 h with the permanent stirring (for better ligand exchange, stirring was applied overnight). The next day, the particles were precipitated with excess hexanes and again diluted in pyridine.

### Characterization

UV/vis spectra were recorded using a Carry 100 absorption spectrophotometer. The samples were dispersed in 1-cm path length quartz cells filled with hexane or pyridine. The absorbance values were used to calculate the molar particle extinction coefficients, the NCs diameters and their concentrations [37].

1H and 31P nuclear magnetic resonance (NMR) spectra were recorded on a Bruker DRX 500 and Bruker Avance III 500 NMR spectrometers operating at 500 and 202.772 MHz, respectively, and referenced to tetramethylsilane at *δ *0.0 or in the scale relative to CDCl_3 _using the residual proton signal of CDCl_3 _at *δ *7.24 in the case of 1H NMR spectra and to an external phosphoric acid standard set at *δ *0.0 in the case of ^31^P NMR spectra. ^1^H NMR and ^31^P NMR analyses were carried out either by dissolving a purified powder of a NC sample in CDCl3 or by using the recovered ligands dissolved in CDCl_3_. To obtain the recovered ligands, purified NCs were first digested by a DCl-D_2_O solution, and then the organic ligands were extracted by CDCl_3 _from the D_2_O solution [38].

High-resolution transmission electron microscopy (TEM) images were collected by using a Philips CM 200 FEG transmission electron microscope operating at an acceleration voltage of 200 kV. A 10 -μl drop from a very dilute sample solution was deposited on a carbon-copper grid and left to evaporate in air.

Powder X-ray diffraction patterns were collected on a X'Pert Pro MPD instrument from PANalytical using Cu K_α _radiation (*λ *= 1.5406 Å) in the range of 5° to 80°. Samples were deposited from a concentrated suspension of the particles on the special low-background silicon substrates at room temperature.

Raman spectra were excited with the 20-mW 488-nm Ar^+ ^laser line (power density on the sample about 400 W/cm^2^) and recorded with a spectral resolution of 2 cm^-1 ^using a triple monochromator Raman system (Dilor XY 800) equipped with a CCD camera. Raman spectra were taken at 50 K, provided by a closed cycle helium cryostat, from the CdTe NCs samples deposited from solution on a Si substrate. The phonon peak of the Si substrate was used as an internal standard for a precise calibration of the phonon frequencies of the CdTe NCs.

## Results and discussion

### Optical absorption

**Figure 1:**
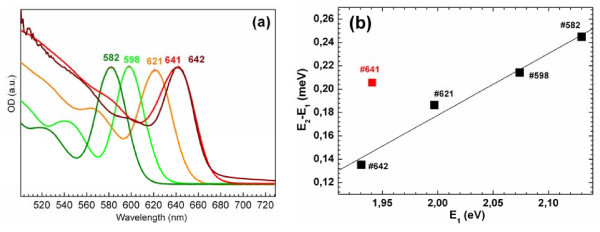
**Optical spectra**. Optical absorption spectra of the oleic acid-stabilized CdTe NCs under study (**a**) and (*E*_2 _- E_1_) splitting vs *E*_1 _(**b**).

The optical absorption spectra of the series of OA-stabilized CdTe NCs under study are shown in Figure 1.

The significant blue shift of the lowest energy absorption peak at 582 to 642 nm (2.00 to 1.93 eV) from the bandgap energy of the bulk CdTe - 1.43 eV [39] - evidences strong confinement of carriers in these NCs. Based on the sizing curves reported in literature [37], the average diameter of the NCs in our samples was estimated to vary from 3.5 to 4.2 nm (Table 1).

**Figure 2:**
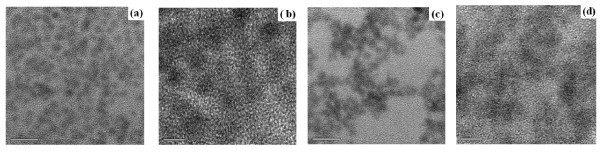
**Images of the TEM**. TEM images of the CdTe NC sample #642 in hexane (**a-b**) and in pyridine (**c-d**). The scale is 20 nm in (a, c) and 5 nm in (b, d).

The distinct lowest energy features in the spectra indicate a narrow size distribution (~5% [40]) and high crystallinity of the NCs, in agreement with the results of TEM (Figure 2a), X-ray and previous investigation of NCs prepared in the similar way [8,40,41], which possess quantum yield of the photoluminescence as high as approximately 80% at room temperature [40]. The NC size determined from TEM correlates well with that calculated from absorption.

At a closer look on the absorption spectra of #641 and #642, one can notice the distance between two lowest absorption peaks, Δ*E*_21 _= *E*_2 _- *E*_1_, being very different - 20 and 40 nm, respectively (Figure 1a). It is seen from the plot of the Δ*E*_21 _vs *E*_1 _energy in Figure 1b that the sample #641 does not follow the trend of the rest of samples. If we assume the same mean NC size for samples #641 and #642, the different Δ*E*_21 _value may be then related to different Cd/Te ratio or to the different crystalline structure of the NCs - zinc blend vs wurtzite. Indeed, for CdSe NCs, the larger ΔE_21 _was reported to be an indication of zinc-blend structure, with small splitting attributed to the wurtzite one [42].

However, it is unlikely that based solely on those data for CdSe we can build a reliable criterion for the crystalline phase of our CdTe NCs based on the value of Δ*E*_21 _because our analysis of many published works on CdTe NCs revealed no unambiguous relation between the Δ*E*_21 _value and crystalline structure. Thus, the Δ*E*_21 _= 110 to 130 meV (30 to 40 nm) corresponds to *d *= 4 nm for NCs of either zinc-blend or wurtzite structures. The X-ray diffraction measurements of our samples #641 and #642 revealed the same, zinc-blend structure. Based on the inherent size dependence of Δ*E*_21 _[43,44], its magnitude of 135 meV (42 nm) for #642 is more reasonable value for 4 nm NCs than 203 meV (60 nm) observed for #641. Therefore, we can conclude that the position of the excitonic features in the absorption spectrum of the NC sample #641 is strongly affected by factor(s) other than quantum confinement effect (i.e. NC size). Probably, the inhomogeneity of the composition over the NC volume plays a role. This problem will be further discussed in the Raman part of the paper.

### NMR

NMR spectroscopy is a well-known method to determine the composition of the ligand shell and distinguish between bound and free ligands, since the signals from atoms located near the surface of the nanoparticles can be shifted, broadened or even disappear completely. To study the composition of the initial ligand shell, we have fulfilled ^1^H and ^31^P NMR analysis. ^31^P NMR analysis detected no phosphorus compound in the sample, despite the fact that TOPTe is present in the synthesis of CdTe NCs. So, one can draw the conclusion that the ligand shell of the CdTe NCs after synthesis is composed only of oleic acid molecules.

**Figure 3:**
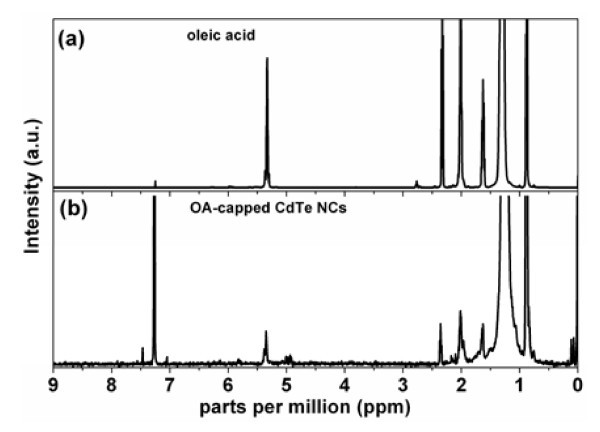
**NMR spectroscopy**. ^1^H NMR spectrum of pure oleic acid (**a**) and recovered ligand shell (**b**) of as-synthesized OA-capped CdTe NCs.

To obtain a clear representation of the initial ligand shell composition of CdTe NCs, we have fulfilled the analysis of the recovered ligands (see experimental section). By comparison of the ^1^H NMR spectra of the recovered ligand shell from the original CdTe NCs (Figure 3b) with pure oleic acid (Figure 3a), one can clearly observe the presence of six typical OA peaks in the nanocrystal sample, i.e. with the same chemical shift as in pure OA. This is in accordance with other studies of OA-capped NCs, where the signals of bound OA did not shift; however, some of the signals were strongly broadened or disappeared [45].

The integrals of the signals do not match these of the pure OA because the signals of the hydrogen atoms located near the surface of the nanoparticles are affected in the strongest way. But taking into account the fact that the presence of TOP in the ligand shell was not detected by means of ^31^P NMR, the only possible ligands of CdTe NCs can be OA molecules.

**Figure 4:**
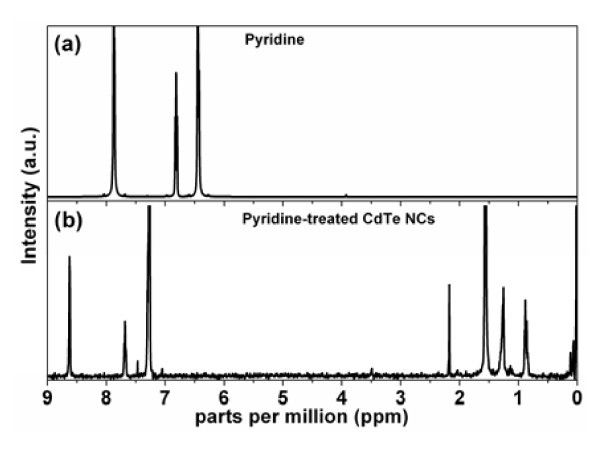
**NMR spectrum**. ^1^H NMR spectrum of (**a**) pure pyridine and (**b**) CdTe NCs after the ligand exchange.

The NMR spectrum of intact CdTe NCs after the pyridine treatment is shown in Figure 4b. Three peaks at 7.30 (overlapped by the signal of CDCl_3_), 7.68 and 8.62 ppm correspond to pyridine, while in the pure pyridine NMR spectrum (Figure 4a) these signals are shifted and are at 6.45, 6.81 and 7.86 ppm, respectively.

The fact that the ligand exchange took place at first view is evidenced by change in the solubility of the particles: As-synthesized NCs dissolve in hexane, but the pyridine-treated NCs precipitate after the addition of hexane and dissolve in pyridine. However, it is known that oleic acid ligands of semiconductor nanoparticles cannot be completely substituted to pyridine ones (even after several repeated steps of the ligand exchange) [46]. Due to the difficulty of complete ligand exchange with pyridine, having a weak binding nature, we assume that some OA molecules are still present in the ligand cap of CdTe NCs.

### Resonant Raman scattering of CdTe NCs of different size

**Figure 5:**
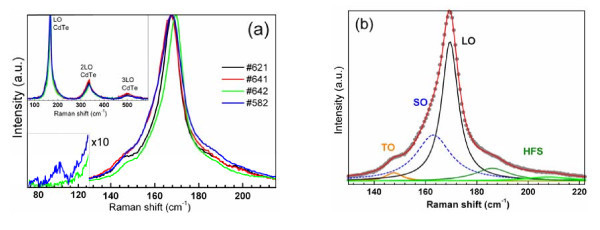
**Raman scattering**. (**a**) Resonant Raman spectra of the CdTe NCs in the range of the LO phonon. The region of the DAP mode ~100 cm^-1 ^is scaled for convenience. Inset shows the spectra in the broad spectral range including LO overtones. (**b**) A sample multi-Lorenzian peak fit of the spectrums in the range of the LO phonon.

Figure 5a shows RRS spectra of the series of initial (OA-stabilized) CdTe NCs in the range of LO phonon (approximately 170 cm^-1^) and its overtones (2LO approximately 340 cm^-1 ^and 3LO approximately 510 cm^-1^), measured at temperature of 50 K and excitation wavelength of 488 nm. The excitation wavelength used falls well above the absorption threshold of the CdTe NCs under study, even at temperature of 50 K at which the absorption spectrum shifts as a whole to shorter wavelength by about 25 nm, providing high enough signal/noise ratio at a moderate excitation power. A care was taken to avoid heating of the NCs, and we observed neither noticeable changes of the intensity and lineshape of the Raman spectrum nor visual damage of the sample, at least at the timescale of spectrum accumulation.

The assignment of the peak at 167 to 169 cm^-1 ^to the scattering on LO phonons in CdTe NC is based on previous observations for CdTe NCs [9-12] and the peak frequency being very close to the LO mode in bulk CdTe crystals - 170 to 173 cm^-1 ^at low temperatures [39]. The difference between the bulk and present values is obviously due to the phonon confinement. The LO peak broadening and low-frequency asymmetry are assigned to phonons with *k *≠ 0 [47], surface optical (SO) [19] or zone-edge (ZE) [18] phonons, or quantized optical vibrations (vibrons) with quantum number *n *> 1 [13]. Even though fitting the spectral curve with two Lorentzians, corresponding to SO (ZE) and LO phonon or to vibrons with different quantum numbers, has been widely applied to CdSe, CdTe and CdS NCs [9,10,17-19,23], for present CdTe NCs the two-peak fit was found to be inappropriate. It is seen from the sample fit in Figure 5b that two Lorentzian profiles are needed to fit satisfactorily only the low-frequency asymmetry of the LO peak. Based on a small peak width and its frequency value matching closely that of the bulk CdTe transverse optical (TO), the lowest component, peaked at 145 cm^-1^, can be assigned to the CdTe TO phonon [11,14]. In contrary to LO, TO mode is not expected to shift noticeably with a reduction of the crystallite size, as the TO dispersion curve of CdTe is rather flat [39]. Note, however, that as the confined optical vibrations (phonons, vibrons) in the NC are of mixed TO-SO-LO nature (except for LO which is purely longitudinal) [13], the assignment of the lowest frequency optical mode to TO can be rather conditional and only mean that this vibrational mode contains more TO nature/movement than other modes.

The broader (Γ = 12 cm^-1^) component peaked near 160 cm^-1 ^(Figure 5b) is likely to be attributed to a 'true' SO modes, as the frequency of the SO mode is expected to lie between the TO and LO [48]. We calculated the *ν*_SO _for our NCs, using the same formalism as in [9], with the low temperature of the present measurements taken into account and the dielectric constant of the ligand shell (*ε*_M _= 2.5). The obtained value of *ν*_SO _= 160.5 cm^-1 ^is very close to that of the experimental component centred at 160 to 162 cm^-1^. A superposition with higher SO modes can lead to the larger width of this feature, as compared to Γ_LO _(6 cm^-1^) and Γ_TO _(5 cm^-1^).

The Raman spectra in Figure 5a follows the common dependence on NC size - the LO band gets broader and shifted downwards when NC size decreases. The only exception is the sample #641 - its broader and downwards shifted LO band does not correspond to the largest size (the same as in #642 - 4.2 nm) in the series, as derived from the *E*_1 _energy in the absorption spectra (Figure 1a, Table 1). It is natural to assume that this finding in the Raman spectra of #641 and the unexpectedly large Δ*E*_21 _value of this sample (Figure 1b) are related to its special structure, different from NC sample #642 and other samples studied. As we mentioned in Section 3.1, the crystalline phase was proved by X-ray to be the same, zinc-blend, for both latter samples. The most probable difference of the #641 can then be a smaller size (compared to #642) due to shorter reaction time and different structures of the surface due to different Cd/Te ratios (Table 1). In this size range, the difference in diameter of several angstroms may hardly be revealed by TEM or X-ray but can result in noticeable difference in absorption spectra. The different Cd/Te ratios as well as the reaction time (Table 1) can readily lead to different structures of the NCs at coinciding *E*_1 _energy. Such competing, with respect to the spectral shifts, factors as surface-induced strain, surface reconstruction and non-stoichiometry can therefore lead to the observed discrepancy in the optical absorption and phonon spectra.

In particular, the effect of CdTe surface enrichment with Te, well-known for bulk CdTe crystals and thin films [49], can be even more probable for NC due to their large surface-to-volume ratio and surface curvature. Thus, the sample #641 can have some radial composition inhomogeneity, with Te enrichment towards surface and therefore a reduced volume of pure (stoichiometric) CdTe. The reasons are the small time precipitation of Cd at its relatively small (compared to other samples) excess in the parental solution (2:1). For #621 obtained at the same precipitation time, the Cd deficiency is, probably, not realized due to higher Cd/Te ratio - 7:1. On the other hand, for #642 with even slightly smaller Cd/Te ratio (1:1), no noticeable composition inhomogeneity occurs due to longer precipitation time - 20 s.

Based on the above arguments, the downwards shifted and broadened LO phonon peak of the #641 can be well explained by a reduced size of the pure (stoichiometric) CdTe phase. The structural inhomogeneity (disorder) of the near-surface region of the NCs can also contribute to the LO peak broadening. A detailed study of ZnS NPs [32] showed that random imperfections of the NC structure such as non-stoichiometry and bond bending can lead to significant deviations of the elastic (and subsequently vibrational) properties over the NC, while the average (effective) strain can be insignificant. Moreover, the calculations of CdSe NC structure performed in [34] revealed a surface reconstruction even for bare particles, with the inward relaxation of the Cd atoms to reduce the energy of the NC, which induces the compressive strain inside NC.

In view of the discussed above, our observations in the optical and vibrational spectra of NCs studied, the relation between the NC size and its optical properties (in particular, position of the lowest exciton state) appears not to be straightforward, and the commonly used way of estimation of the NC size based only on the position of the lowest absorption maximum is to be used with caution. Moreover, a more detailed study of the effect of Cd/Te ratio at different reaction times onto excitonic and phonon spectra is to be undertaken in the future.

The high-frequency shoulder (HFS) of the LO peak, which expands up to 70 to 80 cm^-1 ^above the LO frequency (Figure 5), has been reported by us recently for NCs of different chemical compositions (CdTe vs CdSe), as well as synthesis conditions (temperature, precursors, solvents etc.) and optical spectra (absorption and PL bandwidth, PL QY) [20]. Here, the feature is seen to be more pronounced for smaller CdTe NCs (Figure 5a), in qualitative agreement with observations for CdSe NCs studied in [20]. Among probable origins of the high-frequency shoulder, the contribution of acoustic phonons or surface-induced vibrational modes was assumed [20]. The version of Cd or Se oxide was excluded for HFS in CdSe NCs in [20] due to mismatch of the vibrational frequencies. Similarly, the oxide origin of the HFS in present CdTe NCs can be excluded. More probably, it appears to originate from elemental Te on the NC surface, as discussed in the next section.

The stronger HFS for smallest NCs - sample #582 (Figure **5**a) - qualitatively correlates with appearance of the peak approximately 100 cm^-1^, commonly assigned to a disorder-activated phonon (DAP) mode [18]. We observed this mode to be activated even in high-quality CdSe NCs of sufficiently small size - less than 3 nm [20] - in accordance with earlier report on CdS NCs in glass [13]. Probably, for smaller enough NC (even with perfect internal crystallinity), the surface itself is a defect that perturbs the phonon propagation, resulting in activation of the DAP mode. Based on the absence of the DAP mode in the spectrum of #641, we can additionally confirm the above assumption that the NC size in this sample is not as small as in # 582 and the low frequency and broadening of the LO peak of #641 results from other effects than phonon confinement (e.g. non-stoichiometry).

### Effect of pyridine treatment on Raman spectra of CdTe NCs

**Figure 6:**
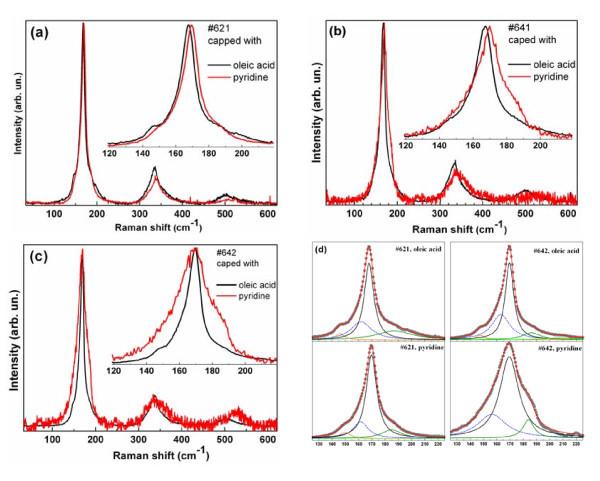
**Pyridine effect on phonon Raman spectrum**. Effect of pyridine on Raman spectra of CdTe NCs: #621 (**a**), #641 (**b**) and #642 (**c**). Fit of the Raman spectra of #621 and #642, which demonstrates the effect of pyridine onto the particular modes (**d**).

The observed effect of pyridine treatment onto the phonon spectra lies generally in the broadening and upwards shift of the main phonon band at approximately 170 cm^-1 ^and its overtones, as well as reduction of the intensity ratio of the overtones to fundamental (Figure 6). The fitting shows that the change in the lineshape in our case is mainly due to broadening of the LO peak, and the magnitude of the enhancement and broadening of its low- and high-frequency shoulders are not as large as it can appear at the first glance. The reason is obviously the much higher intensity of the LO peak compared to other features. The stronger effect of the ligand exchange for NCs #642 than for smaller-sized NCs #621 can be due to discussed in the previous section effect of NC stoichiometry, determined by the Cd/Te ratio in the parental solution. As the ligands are known to bind to Cd but not to Te (or other chalcogens), the sample with smaller Cd/Te ratio (#642 in this case) can reveal a more pronounced effect of the ligand exchange due to smaller number of Cd terminations on the surface of NCs.

Another effect of pyridine treatment is a sharpening and slight downwards shift of the HFS feature. As the enhancement of the HFS after treatment in pyridine was observed for two samples, #641 and #642, with higher Te concentration during synthesis, this Raman feature may be related with the Te phase on the NC's surface. Note that Raman spectroscopy on phonons is an efficient tool to detect the surface Te on CdTe crystalline films [49] due to much larger scattering cross section for Te compared to CdTe [50]. The only reported Te-related Raman feature above CdTe LO phonon frequency is the bond-stretching vibration of the crown-like Te_8 _ring at 182 cm^-1 ^[50], which is also observed in a-Te. Even though the most strong Te-related Raman features, which are more commonly observed, are situated at lower frequencies (90 (*E*), 121 (*A*_1_) and 142 cm^-1 ^(*E*) [50]), these frequencies and width can be significantly affected because of the distortion of Te-Te bonds on the NC surface. It is known that the *A*_1 _mode of Te shifts from 121 cm^-1 ^in bulk c-Te up to 159 cm^-1 ^in a-CdTe due to stronger bonding in the Te chains which lost long-range order (interaction) [51]. When we assume that Te exists in the initial CdTe NC sample, then reduction of the corresponding peak width and its downwards shift to the 182 cm^-1 ^position in the treated sample may be related with improvement of the structural perfection of the Te phase or increase of its volume.

The DAP feature approximately 100 cm^-1^, which can be indicative of surface disorder, was not enhanced or activated by pyridine treatment, and we can conclude that this feature is related rather to small CdTe NC than to Cd/Te ratio because the sample #582, for which this feature is most pronounced, was formed at intermediate Cd/Te ratio (Table 1).

As the surface of the NCs plays an important role in all their properties, the special attention is obviously to be paid to phonon modes traditionally related with the surface. The important property of the SO vibrational mode is that its electric potential exists outside the NC (unlike the strongly localized LO) [48]; thus, it should feel the interparticle interaction better than LO. Indeed, the authors of [12] reported on suppression of the SO mode in array of close-packed CdSe nanorods (NRs). The surface or interface phonon modes are stimulated by the change in dielectric medium at the interface. In a close-packed 2D array of nanorods, the space adjacent to the rods is occupied by other nanorods, and the modified dielectric medium surrounding the individual rods was supposed in [12] to cause the observed suppression of the SO phonon modes. A qualitatively similar response of the SO mode to NC aggregation one could expect for our CdTe NCs after pyridine treatment which is seen to lead to significant aggregation (Figure 2). However, no suppression of the SO mode in our case is observed, even though the NCs are seen on TEM images to be in contact with one another. Obviously, the nature of the SO mode is not well understood at the moment as its behaviour varies strongly from one study to another. The different responses of the SO mode to the close packing of the NCs in our work and in [12] can be explained by an inherent anisotropy of the NRs' properties in [12]. The special properties (e.g. spectral) of the SO mode(s) and their response to certain polarization of the electric field of the Raman-exciting light can thus be induced by the anisotropy of the SO mode in individual NR that allows a collective anisotropy to be induced in the aligned array of close-packed NRs. We believe this factor can be more important than the close packing of the NCs itself ('*ε*-contrast effect') because the packing density of the NCs in the aligned and random samples does not differ much [12].

## Conclusions

The dependence of the phonon spectra of colloidal CdTe NCs on the kind of surface ligand was studied by resonant Raman spectroscopy, NMR, optical absorption and TEM. The exchange of oleic acid molecules on the NC surface for pyridine ones was found to influence noticeably the positions and width of the LO phonon peak, as well as its intensity ratio to overtones. The broad high-frequency shoulder above the LO peak was stronger in smaller NCs and shifted upwards and sharpened after pyridine treatment. The low-frequency mode around 100 cm^-1^, commonly related with the disorder-activated acoustical phonons, appears in small (3.5 nm) NCs but is not enhanced after pyridine treatment. The effect of the Cd/Te ratio in parental solution is found in both the splitting in optical absorption spectra and LO phonon energy and broadening. Surprisingly, the feature at low-frequency side of the LO peak, commonly assigned to the surface optical phonon mode, was not sensitive to ligand exchange and concomitant close NC packing. The observed changes in the phonon spectrum of ligand-exchanged CdTe NCs are related with an increased structural disorder on the NC surface, strain and modified electron-phonon coupling.

## Competing interests

The authors declare that they have no competing interests.

## Authors' contributions

VD carried out the interpretation of the Raman data and drafted the manuscript. IL did the pyridine treatment of nanoparticles and wrote parts of the manuscript. CH carried out acquisition of the Raman. XJ synthesized the nanoparticles. JKO wrote parts of the manuscript. DRTZ have been involved in drafting the manuscript and its revision. All authors read and approved the final manuscript.

## References

[B1] Rogach A (Ed) Semiconductor nanocrystal quantum dots: synthesis, assembly, spectroscopy and applications and the references therein 2008 New York: Springer-Verlag GmbH

[B2] Kinge S Crego-Calama M Reinhoudt DN Self-Assembling Nanoparticles at Surfaces and Interfaces ChemPhysChem 2007 9 20 10.1002/cphc.200700475 18080256

[B3] Kniprath R Rabe JP McLeskey JT Jr Wang D Kirstein S Hybrid photovoltaic cells with II-VI quantum dot sensitizers fabricated by layer-by-layer deposition of water-soluble components Thin Solid Films 2009 518 295

[B4] Fahmi A Pietsch T Appelhans D Gindy N Voit B Water-soluble CdSe nanoparticles stabilised by dense-shell glycodendrimers New J Chem 2009 33 703

[B5] Byrne SJ Williams Y Davies A Corr SA Rakovich A Gun'ko YK Rakovich YP Donegan JF Volkov Y "Jelly Dots": Synthesis and Cytotoxicity Studies of CdTe Quantum Dot-Gelatin Nanocomposites Small 2007 3 1152 10.1002/smll.200700090 17534993

[B6] Kim BS Islam MA Brus LE Herman IP Interdot interactions and band gap changes in CdSe nanocrystal arrays at elevated pressure J Appl Phys 2001 89 8127

[B7] Dias EA Sewall SL Kambhampati P Light Harvesting and Carrier Transport in Core/Barrier/Shell Semiconductor Nanocrystals J Phys Chem C 2007 111 708

[B8] Trotzky S Kolny-Olesiak J Falke SM Hoyer T Lienau C Tuszynski W Parisi J Ligand removal from soluble CdTe nanocrystals evidenced by time-resolved photoluminescence spectroscopy J Phys D Appl Phys 2008 41 102004

[B9] de Paula AM Barbosa LC Cruz CHB Alves OL Sanjurjo JA Cesar CL Size effects on the phonon spectra of quantum dots in CdTe-doped glasses Appl Phys Lett 1996 69 357

[B10] Freire PTC Araújo Silva MA Reynoso VCS Vaz AR Lemos V Pressure Raman scattering of CdTe quantum dots Phys Rev B 1997 55 6743

[B11] Dzhagan V Ya M Valakh Kolny-Olesiak J Lokteva I Zahn DRT Resonant Raman study of phonons in high-quality colloidal CdTe nanoparticles Appl Phys Lett 2009 94 243101

[B12] Nobile C Fonoberov VA Kudera S Della Torre A Ruffino A Chilla G Kipp T Heitmann D Manna L Cingolani R Balandin AA Krahne R Confined Optical Phonon Modes in Aligned Nanorod Arrays Detected by Resonant Inelastic Light Scattering NanoLetters 2007 7 476 10.1021/nl062818+ 17243753

[B13] Rolo AG Vasilevskiy MI Raman spectroscopy of nanostructures and nanosized materials J Raman Spectrosc 2007 38 618

[B14] Vasilevskiy MI Trallero-Giner C Resonant Raman scattering in spherical quantum dots: II-VI versus III-V semiconductor nanocrystals Phys Stat Sol (b) 2010 247 1488

[B15] Lange H Artemyev M Woggon U Thomsen C Optical phonons in colloidal CdSe nanorods Phys Stat Sol (b) 2009 246 2817 2819

[B16] Lu L Xu X-L Liang W-T Lu H-F Raman analysis of CdSe/CdS core-shell quantum dots with different CdS shell thickness J Phys Condens Matter 2007 19 406221 10.1088/0953-8984/19/40/406221 22049120

[B17] Dzhagan VM Valakh MYa Raevskaya OE Stroyuk OL Kuchmiy SYa Zahn DRT The influence of shell parameters on phonons in core-shell nanoparticles: a resonant Raman study Nanotechnology 2009 20 365704 10.1088/0957-4484/20/36/365704 19687558

[B18] Ingale A Rustagi KC Raman spectra of semiconductor nanoparticles: Disorder-activated phonons Phys Rev B 1998 58 7197

[B19] Roy A Sood AK Surface and confined optical phonons in CdSxSe1-x nanoparticles in a glass matrix Phys Rev B 1996 53 12127 10.1103/physrevb.53.12127 9982841

[B20] Dzhagan VM Lokteva I Valakh MYa Raevska OE Kolny-Olesiak J Zahn DRT Spectral features above LO phonon frequency in resonant Raman scattering spectra of small CdSe nanoparticles J Appl Phys 2009 106 084318

[B21] Kelley AM Electron-Phonon Coupling in CdSe Nanocrystals J Phys Chem Lett 2010 1 1296

[B22] Yaremko AM Yukhymchuk VO Valakh MYa Pascual J Novikov AV Mozdor EV Mestres N Lytvyn PM Krasilnik ZF Klad'ko VP Dzhagan VM Theoretical and experimental investigations of single- and multylayer structures with SiGe nanoislans Mater Sci Eng C 2003 23 1027

[B23] Azhniuk YuM Milekhin AG Gomonnai AV Lopushansky VV Turok II Yukhymchuk VO Zahn DRT Phonon spectra of quaternary Cd1-yZny S1-xSex semiconductor nanocrystals grown in a glass matrix Phys Status Solidi (a) 2004 201 1578

[B24] Meulenberg RW Jennings T Strouse GF Compressive and tensile stress in colloidal CdSe semiconductor quantum dots Phys Rev B 2004 70 235311

[B25] Melnik NN Sadofyev YuG Zavaritskaya TN Vodop'yanov LK Multiphonon relaxation in ZnSe thin films and ZnSe/ZnCdSe MQW structures Nanotechnology 2000 11 252

[B26] Reparaz JS Bernardi A Goni AR Lacharmoise PD Alonso MI Garriga M Novak J Vavra I Phonon pressure coefficient as a probe of the strain status of self-assembled quantum dots Appl Phys Lett 2007 91 081914

[B27] Lange H Artemyev M Woggon U Thomsen C Geometry dependence of the phonon modes in CdSe nanorods Nanotechnology 2009 20 045705 10.1088/0957-4484/20/4/045705 19417331

[B28] Shiang JJ Risbud SH Alivisatos AP Resonance Raman studies of the ground and lowest electronic excited state in CdS nanocrystals J Chem Phys 1993 98 8432

[B29] Chassaing P-M Demangeot F Paillard V Zwick A Combe N Pagès C Kahn ML Maisonnat A Chaudret B Surface optical phonons as a probe of organic ligands on ZnO nanoparticles: An investigation using a dielectric continuum model and Raman spectrometry Phys Rev B 2008 77 153306

[B30] Azhniuk YuM Milekhin AG Gomonnai AV Hutych YuI Lopushansky VV Zahn DRT Phonon spectra of quaternary Cd1-yZny S1-xSex semiconductor nanocrystals grown in a glass matrix Phys Stat Sol (c) 2009 6 2068

[B31] Sun Z Zhao B Lombardi JR ZnO nanoparticle size-dependent excitation of surface Raman signal from adsorbed molecules: Observation of a charge-transfer resonance Appl Phys Lett 2007 91 221106

[B32] Gilbert A Zhang H Chen B Kunz M Huang F Banfield JF: Compressibility of zinc sulfide nanoparticles Phys Rev B 2006 74 115405

[B33] Huxter VM Lee A Lo SS Scholes GD CdSe Nanoparticle Elasticity and Surface Energy NanoLett 2009 9 405 10.1021/nl803275a 19061357

[B34] Puzder A Williamson AJ Gygi F Galli G Self-Healing of CdSe Nanocrystals: First-Principles Calculations Phys Rev Lett 2004 92 217401 10.1103/PhysRevLett.92.217401 15245315

[B35] Xu S Wang C Cui Y Theoretical investigation of CdSe clusters: influence of solvent and ligand on nanocrystals J Mol Model 2010 16 469 10.1007/s00894-009-0564-4 19636596

[B36] Zutz F Lokteva I Radychev N Kolny-Olesiak J Riedel I Borchert H Parisi J Study of the influence of the Cd:Se precursor ratio during the synthesis of CdSe nanocrystals on the performance of CdSe/P3HT hybrid solar cells Phys Stat Solidi (a) 2009 206 2700

[B37] de Mello Doneg C Koole R Size Dependence of the Spontaneous Emission Rate and Absorption Cross Section of CdSe and CdTe Quantum Dots J Phys Chem C 2009 113 6511

[B38] Yu WW Wang YA Peng X Formation and Stability of Size-, Shape-, and Structure-Controlled CdTe Nanocrystals: Ligand Effects on Monomers and Nanocrystals Chem Mater 2003 15 4300

[B39] Madelung O Rössler U Schulz M Physics of II-VI and I-VII compounds, semimagnetic semicond., Landolt-Bornstein, new series, group III 1982 41 Pt B Berlin: Springer

[B40] Kloper V Osovsky R Kolny-Olesiak J Sashchiuk A Lifshitz E The Growth of Colloidal Cadmium Telluride Nanocrystal Quantum Dots in the Presence of Cd0 Nanoparticles J Phys Chem C 2007 111 10336

[B41] Kolny-Olesiak J Kloper V Osovsky R Sashchiuk A Lifshitz E Synthesis and characterization of brightly photoluminescent CdTe nanocrystals Surf Sci 2007 601 2667

[B42] Mohamed MB Tonti D Al-Salman A Chemseddine A Chergui M Synthesis of high quality zinc blende CdSe nanocrystals J Phys Chem B 2005 109 10533 10.1021/jp051123e 16852276

[B43] Efros AL Rosen M Quantum size level structure of narrow-gap semiconductor nanocrystals: Effect of band coupling Phys Rev B 1998 58 7120

[B44] Zhong H Nagy M Jones M Scholes GD Electronic states and exciton fine structure in colloidal CdTe nanocrystals J Phys Chem C 2009 113 10465

[B45] Ji X Copenhaver D Sichmeller C Peng X Ligand Bonding and Dynamics on Colloidal Nanocrystals at Room Temperature: The Case of Alkylamines on CdSe Nanocrystals J Am Chem Soc 2008 130 5726 10.1021/ja710909f 18396878

[B46] Lokteva I Radychev N Witt F Borchert H Parisi J Kolny-Olesiak J Surface Treatment of CdSe Nanoparticles for Application in Hybrid Solar Cells: The Effect of Multiple Ligand Exchange with Pyridine J Phys Chem C 2010 114 12784

[B47] Campbell IH Fauchet PM The effects of microcrystal size and shape on the one phonon Raman spectra of crystalline semiconductors Solid State Commun 1986 58 739

[B48] Hayashi S Kanamori H Raman scattering from the surface phonon mode in GaP microcrystals Phys Rev B 1982 26 7079

[B49] Artamonov VV Valakh MYa Strel'chuk VV Baidullaeva A Mozol' PE Raman scattering by tellurium films on CdTe single crystals J Appl Spectroscopy 1988 48 653

[B50] Poborchii VV Raman spectra of sulfur, selenium or tellurium clusters confined in nano-cavities of zeolite A Solid State Commun 1998 107 513

[B51] Morell G Reynés-Figueroa A Katiyar RS Farías MH Espinoza-Beltran FJ Zelaya-Angel O Sánchez-Sinencio F Raman spectroscopy of oxygenated amorphous CdTe films J Raman Spectr 1994 25 203

